# Malaria case clinical profiles and *Plasmodiumfalciparum* parasite genetic diversity: a cross sectional survey at two sites of different malaria transmission intensities in Rwanda

**DOI:** 10.1186/s12936-016-1287-5

**Published:** 2016-04-26

**Authors:** Fredrick Kateera, Sam L. Nsobya, Stephen Tukwasibwe, Petra F. Mens, Emmanuel Hakizimana, Martin P. Grobusch, Leon Mutesa, Nirbhay Kumar, Michele van Vugt

**Affiliations:** Medical Research Centre Division, Rwanda Biomedical Centre, PO Box 7162, Kigali, Rwanda; Division of Internal Medicine, Department of Infectious Diseases, Centre of Tropical Medicine and Travel Medicine, Meibergdreef 9, 1100 DD Amsterdam, The Netherlands; Molecular Research Laboratory, Infectious Disease Research Collaboration, New Mulago Hospital Complex, PO Box 7051, Kampala, Uganda; Department of Pathology, School Biomedical Science, College of Health Science, Makerere University, PO Box 7072, Kampala, Uganda; Royal Tropical Institute/Koninklijk Instituutvoor de Tropen, KIT Biomedical Research, Meibergdreef 39, 1105 AZ Amsterdam, The Netherlands; School of Medicine, College of Medicine and Health Sciences, University of Rwanda, PO Box 3286, Kigali, Rwanda; Department of Tropical Medicine, School of Public Health and Tropical Medicine, Vector-Borne Infectious Disease Research Centre, Tulane University, 333 S Liberty Street, Mail code 8317, New Orleans, LA 70112 USA

**Keywords:** Malaria, *Plasmodiumfalciparum*, Parasite density, Multiplicity of infection, Rwanda

## Abstract

**Background:**

Malaria remains a public health challenge in sub-Saharan Africa with *Plasmodiumfalciparum* being the principal cause of malaria disease morbidity and mortality. *Plasmodium falciparum* virulence is attributed, in part, to its population-level genetic diversity—a characteristic that has yet to be studied in Rwanda. Characterizing *P. falciparum* molecular epidemiology in an area is needed for a better understand of malaria transmission and to inform choice of malaria control strategies.

**Methods:**

In this health-facility based survey, malaria case clinical profiles and parasite densities as well as parasite genetic diversity were compared among *P.falciparum*-infected patients identified at two sites of different malaria transmission intensities in Rwanda. Data on demographics and clinical features and finger-prick blood samples for microscopy and parasite genotyping were collected^.^ Nested PCR was used to genotype *msp*-*2* alleles of FC27 and 3D7.

**Results:**

Patients’ variables of age group, sex, fever (both by patient report and by measured tympanic temperatures), parasite density, and bed net use were found differentially distributed between the higher endemic (Ruhuha) and lower endemic (Mubuga) sites. Overall multiplicity of *P.falciparum* infection (MOI) was 1.73 but with mean MOI found to vary significantly between 2.13 at Ruhuha and 1.29 at Mubuga (p < 0.0001). At Ruhuha, expected heterozygosity (E_H_) for FC27 and 3D7 alleles were 0.62 and 0.49, respectively, whilst at Mubuga, E_H_ for FC27 and 3D7 were 0.26 and 0.28, respectively.

**Conclusions:**

In this study, a higher geometrical mean parasite counts, more polyclonal infections, higher MOI, and higher allelic frequency were observed at the higher malaria-endemic (Ruhuha) compared to the lower malaria-endemic (Mubuga) area. These differences in malaria risk and MOI should be considered when choosing setting-specific malaria control strategies, assessing *p. falciparum* associated parameters such as drug resistance, immunity and impact of used interventions, and in proper interpretation of malaria vaccine studies.

## Background

In spite of the significant decline in malaria cases and deaths being reported globally, malaria still accounted for about 200 million cases and over 500,000 deaths in 2014 [1]. The malaria burden decline, particularly in sub-Saharan Africa, has been associated with the rapid scaling-up of interventions including long-lasting insecticide-treated nets (LLINs), indoor residual spraying (IRS) with insecticides, and use of artemisinin-based combinational therapy (ACT) for managing uncomplicated malaria cases [2]. Scale-up of LLINs, IRS and ACT implementation in Rwanda was associated with a more than 50 % decline in malaria morbidity and mortality among children aged under 5 years between 2005 and 2010 [3]. In spite of these declines however, malaria is still a public health challenge with the entire Rwandese population considered as being at risk.

Human malaria infections exhibit a broad clinical spectrum ranging from asymptomatic infection to severe life-threatening disease. Disease severity is influenced by interactions between parasite, human host and environmental factors, including, but not limited to, anti-malaria therapies used, levels of immunity, age, sex, and pregnancy status [4]. With regard to anti-malaria therapies in Rwanda, resistance in *Plasmodiumfalciparum* in the past led to chloroquine being replaced with amodiaquine (AQ) + sulfadoxine–pyrimethamine (SP) in 2001 and then later, AQ + SP combination was subsequently replaced with artemether–lumefantrine (AL) in 2006, as first-line anti-malarial therapies for uncomplicated malaria. Malaria transmission levels and the associated risk of morbidity and mortality show a spatial heterogeneity even within small countries such as Rwanda [5, 6]. Current Rwandan malaria heterogeneity is partly influenced by the variations in type and intensity of malaria control interventions deployed across different settings as well as the baseline residual transmission potentials at the four different malaria transmission zones [5]. Understanding malaria disease severity, including clinical features and parasitaemia levels associated with malaria disease, in populations from areas of differing malaria transmission intensities is needed for decision making on which control tools may have optimal impact.

*Plasmodium falciparum* is the most prevalent cause of malaria morbidity and mortality in Rwanda [5]. *Plasmodiumfalciparum* virulence is mediated, in part, by its population-level genetic diversity which has been reported to influence malaria disease pathology [7–9], acquisition of immunity [10, 11], infection transmission intensity [12–14], and vaccine responses [15, 16]. High malaria-endemic areas tend to have extensive *P. falciparum* genetic diversity with infected humans often found with multiple genotypes. Conversely, low transmission areas tend to yield limited *P. falciparum* genetic diversity with a higher proportion of infections being monoclonal [17–20].

Studying plasmodial molecular epidemiology is essential to understanding malaria transmission. Currently, malaria disease severity among health facility-identified cases as well as population-level parasite diversity remains unknown in Rwanda. This study compared clinical profiles of malaria-confirmed cases, parasite densities and *P.falciparum* genetic diversity [21, 22] based on the *msp*-*2* gene—a valid, reliable and highly discriminatory and polymorphic marker used for genetic finger printing, at two sites of differing malaria transmission intensities in Rwanda.

## Methods

### Study design and sites

**Fig. 1:**
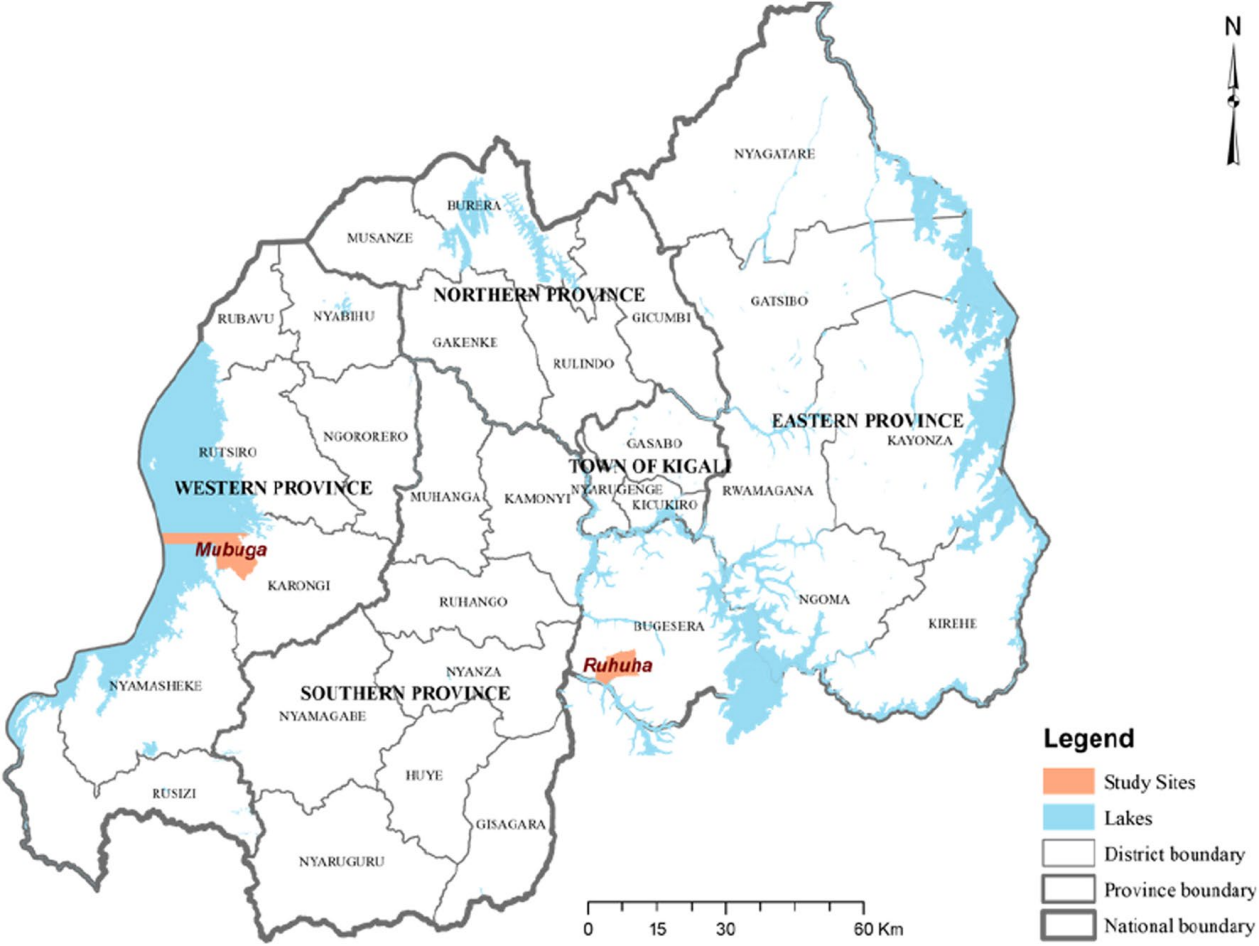
Location map showing study sites of Ruhuha^#^ and Mubuga^#^ sectors in Rwanda. ^**#**^Ruhuha sector is located in Bugesera District, Eastern Rwanda whilst Mubuga sectors is located in Karongi District, Western Rwanda

Rwanda is divided into four malaria ecologic zones based on altitude, climate, level of transmission, and disease vector prevalence [5]. Malaria cases for this cross-sectional survey were recruited from rural Ruhuha sector (Bugesera District, Eastern Province) and Mubuga sector (Karongi District, Western Province) (Fig. 1) located in the highest and lowest malaria transmission zones, respectively [5].

### Baseline demographics, clinical features and blood sample collection

All health facility-visiting cases aged ≥6 months who were microscopically confirmed by the health facility laboratory technicians to be *P*. *falciparum* infected in the period 15th January to 15th February 2015 were eligible for enrolment. Upon provision of written informed consent, a brief structured questionnaire was administered to collect data on demographics (sex, age, area of residence), fever history, and bed net use on the night before the survey. In addition, body temperature was measured using an electronic tympanic thermometer and a second round of finger-prick blood samples taken off to prepare thick and thin smears for analysis by the study laboratory technicians and for blotting on to filter papers (Whatman 3MM) for use in performing molecular studies.

### Preparation of blood films, microscopic examination and quality assurance

Thick blood smears were stained with 3 % Giemsa for 60 min and slides read by two blinded study microscopists. In case of two discordant results, a third reader was used to resolve the discrepancy. Using the thick blood smear, parasite densities were enumerated as the number of counts of asexual parasites per 200 leukocytes, assuming a median leukocyte count of 8000/μL. Thin smears were used to differentiate *Plasmodium* species. External quality control was done on a 10 % sample of randomly selected thick and thin smears by microscopists at the National Reference Laboratory, Kigali, Rwanda whose results were in agreement with those reported by the study technicians.

### *Plasmodium falciparum* DNA extraction and *msp*-*2* allelic typing

DNA was extracted with Chelex 100 Resin (Bio-Rad Laboratories, Hercules, CA, USA) as previously described [23]. The surface antigen loci *msp*-*2* was amplified using previously described primers [24]. Briefly, 2 μL of template DNA was amplified using nested polymerase chain reaction (nPCR), with second-round primers specific to *msp*-*2* allelic families. PCR products were then separated on a 2.5 % agarose gel (UltraPure Agarose; Invitrogen, Carlsbad, CA, USA) and stained with ethidium bromide. GelCompar II software (Applied Maths, Sint-Martens-Latem, Belgium) was used to select alleles and estimate PCR product size as described elsewhere [21].

### Statistical analysis

Demographics, clinical features and bed net use data were collected using hard copy study case record forms while laboratory results were transcribed into study laboratory registers. Both datasets were double-entered into EPI Info™ 7 (Centres for Disease Control and Prevention, GA, USA) database and later transferred into STATA (version 13.1, College Station, TX, USA) for analysis. Parasitaemia—the number of parasites/μL was graded as low (<1000), moderate (1000–9999) and high (>10,000) as per WHO parasitaemia cut-off for severe malaria in low transmission settings [25]. MOI was defined as the proportion of people who carry more than one allele (genotype) for any of the examined genes. Mean multiplicity of infection (MOI) was estimated by dividing the total number of distinct *msp2* genotypes detected by the number of positive samples.

Descriptive statistics of proportions and means were used to summarize distributions of allelic families, baseline demographics, MOI, and other covariate data. Chi square tests were used to compare mean MOI and allelic variant distributions between study sites. Independent t test was used to compare mean MOI outcome by independent factors of age group, study site, history of fever and presence of measured fever (≥37.5 °C), sex and bed net use. Expected heterozygosity index (HE)—which measures locus diversity—was calculated using the formulae HE = [n/(n–1)] [(1–!P_i_^2^)], where n = sample size, Pi = allelic frequency. Odds ratios (ORs) and 95 % confidence intervals (CIs) were calculated to evaluate the strengths of associations. Statistical significance was defined as p value ≤ 0.05 [22].

### Ethical clearance

All adults and carers of children <18 years old were informed of the purpose and procedures of the study, and recruited only after obtaining informed written consent. The study was approved by the National Health Research Committee and the Rwanda National Ethics Committee (No. 20/RNEC/2015), Kigali, Rwanda.

## Results

### Baseline study participant demographics

A total of 407 patients who were microscopically confirmed with malaria by health facility laboratory technicians were enrolled. Of these, 402 (98.8 %) were microscopically re-confirmed by study-trained technicians to be malaria positive. Of the 402, final data analysis was performed on 388 (96.5 %) who were successfully genotyped for the *msp*-*2* alleles. Stratified by site, 195 (50.3 %) cases were enrolled at Ruhuha whilst 193 (49.7 %) enrolled at Mubuga (Table 1). A higher proportion (55.4 %) of study participants were females. The overall group mean age was 15.5 years (SD ± 13.6). Overall geometric mean parasite density was 1119.3 parasites/μL.

**Table 1:** Demographics, malaria prevention, clinical profiles and geometric mean parasite densities/*μL for malaria cases identified in Ruhuha and Mubuga sites in Rwanda

Variables		Ruhuha site n = 195	Mubuga site n = 193	Pearson’s χ^2^ test
Demographics	Variable sub-groups	n (%)	n (%)	
Age groups	6 months–5 years	52 (26.7)	22 (11.4)	–
	6–15 years	95 (48.7)	93 (48.2)	–
	16–73 years	48 (24.6)	78 (40.4)	<0.0001
Sex	Males	77 (39.5)	96 (49.7)	–
	Females	118 (60.5)	97 (50.3)	0.042
Malaria prevention used	Number reporting bed net use night prior to survey	129 (66.2)	150 (77.7)	0.011
Fever history and experience	Number with history of fever in previous 24 h	192 (98.5)	162 (83.9)	<0.0001
	Number with tympanic temperature of ≥37.5 °C	78 (41.5)	110 (58.5)	0.001
Parasitology	Parasite count ranges/per μL Low (<1000)	63 (32.3)	113 (58.6)	
	Moderate (1000–9999)	58 (29.7)	74 (38.3)	
	Severe (≥10,000)	74 (38.0)	6 (3.1)	<0.0001
	Geometric mean parasitaemia (parasites/μL)	2347.3 (95 % CI: 1772.1–3109.2)	529.7 (95 % CI: 402.3–697.4)	<0.0001

*χ* ^2^ chi square test

### Demographics, clinical features, parasitological and malaria control use distributions among participants from the two study sites

The results of group comparisons of demographic, bed net use, fever experiences, and parasite density among patients from the two study sites are shown in Table 1. Significant differences in proportions of participant characteristics of sex (p = 0.04) and age group (p = <0.0001) between patients from Ruhuha and Mubuga sites were noted. At Ruhuha, a higher proportion (60.5 %) of patients were females compared to Mubuga (50.3 %). Among children aged <5 years, a higher proportion was seen at Ruhuha (26.7 %) compared to Mubuga (11.4 %) while among those aged >15 years, a higher proportion was enrolled at Mubuga (40.4 %) compared to Ruhuha (24.6 %). With regard to history of reported fever, a significantly (p = 0.001) higher proportion (99 %) was noted at Ruhuha compared to that reported at Mubuga (84 %). In contrast, a significantly higher proportion of patients (p = 0.001) with a measured temperature of ≥37.5 °C were seen at Mubuga (58.5 %) compared to that reported from Ruhuha (41.5 %). A significantly higher proportion (38.0 %) of patients at Ruhuha had high parasite count (>10,000 parasites/μL) than those seen at Mubuga (3.1 %; p < 0.0001). Similarly, geometric mean parasitaemia counts were higher at Ruhuha (95 % CI: 5686.5–7394.8) than at Mubuga (95 % CI: 1383.3–2251.7). Bed net use was significantly higher at Mubuga (77.7 %) than at Ruhuha (66.2 %) (p = 0.001).

### Infection clones and allelic diversity

Overall, a range of one to six infection clones per sample was seen. At both sites, about 55.4 % of the infections were monoclonal, with isolates from the Mubuga site carrying a significantly higher proportion of monoclonal infections (73 %) compared to those from Ruhuha (38 %) (p < 0.0001). The numbers of strains per isolate are presented in Table 2. Overall, a total of 80 (27.8 %) samples were co-infected by both FC27 and 3D7 types but with the number of strains per isolate noted to be higher at Ruhuha (p < 0.0001) compared to Mubuga (Table 2). In total, more 3D7 allelic variants were detected (298) compared to FC27 variant (184) alleles.

**Table 2:** *Plasmodium falciparum* *msp*-*2* PCR product numbers, size by base pair range and H_E_ for isolates with ≥one allele identified

Variable characteristic	Variable sub-group	Ruhuha n (%)	Mubuga n (%)
Number of clones per sample	1	74 (38.0)	141 (73.0)
	2	60 (30.8)	48 (24.9)
	3	35 (17.9)	3 (1.6)
	4	18 (9.2)	1 (0.5)
	5	3 (1.5)	0 (0.0)
	6	5 (2.6)	0
*msp*-*2* strain distribution			
3D7 strain^a^	0	33 (16.9)	65 (33.7)
	1	93 (47.7)	122 (63.2)
	2	53 (27.2)	5 (2.6)
	3	15 (7.7)	1 (0.3)
	4	1 (0.5)	0 (0.0)
FC27 strain^b^	0	78 (40.0)	86 (44.6)
	1	86 (44.1)	99 (51.3)
	2	15 (7.7)	8 (4.2)
	3	11 (5.6)	0 (0.0)
	4	5 (2.6)	0 (0.0)
PCR products per base pair range			
FC27	300–330	95 (51.9)	114 (85.1)
	350–380	58 (31.4)	17 (12.7)
	400–430	15 (8.1)	2 (1.5)
	450–600	16 (8.6)	1 (0.7)
	Total FC27 PCR products	184	134
	HE average HE (0.44)	0.62	0.26
3D7	200–300	166 (55.7)	120 (83.3)
	320–400	132 (44.3)	22 (16.7)
	Total 3D7 PCR products	298	144
	HE average HE (0.39)	0.49	0.28

^a^3D7 strain difference in distribution = χ^2^ < 0.0001

^b^FC27 strain difference in distribution = χ^2^ = 0.001

### Allelic frequency and heterozygosity

**Fig. 2:**
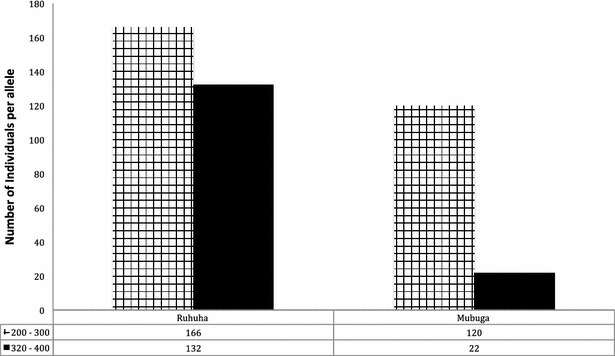
Distribution of *msp*-*2* 3D7 alleles across Ruhuha and Mubuga study sites in Rwanda

**Fig. 3:**
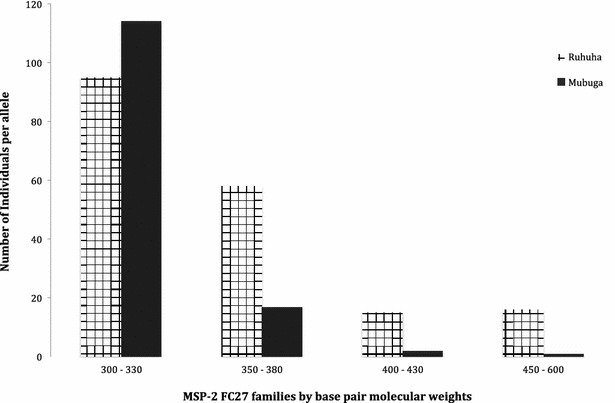
Distribution of *msp*-*2* FC27 alleles between Ruhuha and Mubuga study sites, Rwanda

For both FC27 and 3D7 alleles, 760 distinct *P. falciparum* clones were detected (Table 2). Parasite allelic frequency varied among isolates from the two study sites (Figs. 2, 3). Overall, the majority (68 %) of isolates carried the FC27 300–330-bp size fragment (Fig. 3) while 70 % carried the 3D7 200–300-bp size fragment (Fig. 2). At Ruhuha, HE for 3D7 and FC27 was 0.49 and 0.62 while at Mubuga, HE for 3D7 and FC27 was 0.28 and 0.26, respectively (Table 2). At each of the 3D7 (Fig. 2) and FC27 (Fig. 3) alleles, higher levels of polymorphisms were seen among isolates from Ruhuha than isolates from Mubuga.

### Multiplicity of infection

Results for determinants of MOI are shown in Table 3. Overall, MOI for all infections at both sites was ~1.7. However, MOI varied significantly (*p* < 0.0001) between Mubuga (1.3) and Ruhuha (2.1). In this study, MOI was seen to increase proportional to age group being from 1.7 among those under 5 years old to 1.9 among those aged 6–15 years and 1.5 among those >15 years. Isolates from Ruhuha also had higher MOI compared to those from Mubuga.

**Table 3:** Bivariate analysis for covariate determinants of multiplicity of infection (MOI)

Variable	Variable sub-group	n (%)	MO1 Ruhuha, n = 195	MOI Mubuga, n = 193	Overall MOI, (± SD)	*p* value
Study site	All	388 (100 %)	2.13	1.29	1.72 (± 1.02)	<0.0001
Study participants age group	≤5 years	74 (19.1)	1.92	1.32	1.74 (± 1.05)	
	6–15 years	188 (48.4)	2.34	1.37	1.86 (± 1.07)	
	≥16 years	126 (32.5)	1.96	1.21	1.49 (± 0.89)	0.008
Sex	Male	173 (44.6)	2.14	1.27	1.66 (± 0.97)	
	Female	215 (55.4)	2.13	1.32	1.76 (± 1.06)	0.321
Measured fever ≥37.5 °C	Yes	188 (48.4)	2.12	1.29	1.79 (± 1.10)	
	No	200 (51.6)	2.15	1.30	1.63 (± 0.92)	0.119
Reported fever	Yes	354 (91.2)	2.14	1.29	1.75 (± 1.05)	
	No	34 (8.8)	2.00	1.32	1.38 (± 0.65)	0.046
Parasite density (parasites/μL)	<1000	176 (45.4)	1.91	1.28	1.51 (± 0.82)	
	1000-9999	132 (34.0)	2.39	1.31	1.79 (± 1.10)	
	≥10,000	80 (20.6)	2.12	1.33	2.06 (± 1.18)	0.0002
Number of *Plasmodium* species	*P.* *falciparum* only	215 (55.4)	2.33	1.11	1.73 (± 1.03)	
	*P.* *falciparum* and *P.* *ovale*)	173 (44.6)	2.13	1.30	1.42 (± 0.67)	0.303
Presence of gametocyte	Yes	10 (2.8)	1.50	1.38	1.30 (± 0.56)	
	No	378 (97.2)	2.14	1.29	1.72 (± 1.03)	0.322
History of sleeping under a bed net the night before survey	Yes	279 (71.9)	2.21	1.28	1.71 (± 1.09)	
	No	109 (28.1)	1.99	1.35	1.73 (± 0.84)	0.834

*MOI* multiplicity of infection; *χ* ^*2*^ chi square test; *SD* standard deviation

## Discussion

This study reports, for the first time in Rwanda, a differential spatial distribution of patient demographics of age and sex, fever, parasite density and *P.falciparum* genetic diversity across two study sites. A higher geometrical mean parasite counts (2347 vs 530 parasites), more polyclonal infections, higher MOI and higher allelic frequency were observed at higher malaria-endemic Ruhuha compared to the lower malaria-endemic Mubuga area.

A higher proportion of children aged <5 years was enrolled at Ruhuha compared to Mubuga while, in contrast, a higher proportion of those aged >15 years was recruited at Mubuga compared to Ruhuha. Higher malaria burden in younger age groups in settings of high malaria transmission intensity have been reported previously [26–28]. The age-related association of disease severity across different malaria transmission zones is currently poorly elucidated particularly in the era of scaled-up interventions such as LLINs and IRS whose impact on reducing malaria transmission has also influenced age-related malaria risk. As reported elsewhere scale-up of LLINs has been done [29–32], this study provides further evidence of a shift towards higher malaria risk in older age groups. Results from this study may be confounded by the age-distribution differences between the two sites, with the higher malaria-endemic Ruhuha sector having a higher proportion of sick children aged <5 years. A higher risk of *P.falciparum* infection among younger age groups has been reported from elsewhere, particularly for severe malaria [33]. The apparent higher risk of malaria among younger age groups at the higher endemic Ruhuha site was probably due to a lower clinical protective immunity among the younger age group (<5 years) relative to older age groups (6–15 and >15 years) who may have a higher degree of partially protective immunity already in high transmission settings. In contrast, where malaria control activities, particularly LLIN usage, were scaled up, malaria risk has been observed to shift to older age groups for reasons including delays in acquiring immunity and less bed net use among the older age groups of 6–15 years, compared to children <5 years. A spatial and temporal analysis of changing transmission intensities may provide clarity on allelic frequency epidemiology as determinants of setting-specific malaria risk.

Among patients enrolled at Ruhuha, a significantly higher proportion were females in contrast to those recruited at Mubuga where both sexes were proportionally represented. The association between malaria risk and sex remains equivocal. In contrast to this study’s findings, at the Ruhuha site, a number of previous studies, including two conducted at the Ruhuha site, reported a bias towards higher malaria risk among males [31, 32, 34, 35]. The observed higher proportion of females at Ruhuha in this study may be a chance occurrence due to the non-randomized study design used. In addition, females, as seen in Rwanda, tend to have better health-seeking behaviour, including more frequent visits to health facilities and are more likely to be recruited in health system-based studies than their male counterparts. This is the most probable reason for findings reported here, particularly given that it has been previously established that males had a higher malaria risk in Ruhuha compared to females [31, 34].

In this study, the proportion of patients with a reported fever experiences and by a fever ≥37.5 °C differed across the two sites. Whilst a higher proportion of Ruhuha-recruited patients self-reported a history of fever in the last 24 h compared to those from Mubuga and, in contrast, a lower proportion of the same patients from Ruhuha were confirmed with a measured fever (tympanic temperature ≥37.5 °C) compared to Mubuga patients. Fever is a common malaria-associated symptom and a major reason for seeking care among suspected malaria in endemic settings. At the higher malaria-endemic Ruhuha site, it is plausible that residents are more likely to associate fever with malaria and hence the higher proportion of reported fevers. On the other hand, at the lower malaria-endemic Mubuga site, with presumably a lower proportion of individuals with partially protective levels of immunity, patients are more likely to have symptomatic malaria infections presenting with fever than those at Ruhuha. However, the higher proportion of children <5 years old in Ruhuha may have confounded the observed higher proportion of reported fevers in Ruhuha compared to Mubuga with malaria being associated with fever or recent history of fever in infants. In contrast, the higher malaria endemicity in Ruhuha may plausibly be associated with higher levels of protective immunity leading to a lower proportion of malaria compared to persons from the lower-endemic Mubuga site, as previously reported from the Ruhuha site [31, 34]. Characterizing the association between fever experiences and malaria risk is complicated by other determinants of measured fevers, including population access to and use of antipyretic medications prior to visiting a health facility.

In this study, mean MOI was significantly higher at the higher malaria-endemic Ruhuha site compared to the lower malaria-endemic Mubuga site. While many studies have reported comparable findings of higher MOI in higher endemic settings and correspondingly lower MOI in low endemic settings [17, 20, 36, 37], a study in Ghana did not find any association between MOI and transmission intensity [38]. A plausible reason for higher MOI in higher endemic settings may be the greater diversity and the more frequent meiotic recombination in higher malaria transmission settings. In this study, MOI was noted to significantly decrease with increasing age. Previous studies on associations between MOI and age groups have shown mixed findings, with some reporting no association [36, 39, 40], while others have reported comparable findings of lower MOI with increasing age have been demonstrated in Nigeria, Ghana and Senegal as seen in this study [11, 38, 41]. In a Tanzanian study among children, MOI peaked among those aged 3–7 years suggesting that younger age groups (<10 years) may be contributing significantly to driving parasite diversity [42]. A possible reason for the conflicting findings to those in this study may include differences in study age groups and study site malaria intensities. It is plausible that multiple strains are needed to develop immunity in younger children and hence the higher diversity in younger children. Contrastingly, pre-existing immunity in older age groups may be selectively clearing out some strain types and hence the noted inverse association between MOI and age.

In this study, MOI was positively correlated with parasite density. This finding accords with previous studies where higher MOI among individuals with higher parasite densities has been demonstrated [11, 43]. In contrast, no association between MOI and level of parasitaemia was reported elsewhere [36]. Because parasite densities are influenced by multiple determinants including age, levels of exposure to malaria infections and area-specific transmission levels, these latter factors may partially—either individually or collectively—account for the lack of MOI and parasitaemia level associations observed elsewhere.

About 55 % of the *P.falciparummsp*-*2* confirmed isolates carried monoclonal (single allele) infection. By study site, a higher proportion of monoclonal infections were seen at Mubuga (73.1 %) compared to Ruhuha (38.0 %). These data are similar to other studies where higher proportions of >50 % and up to 100 % polyclonal infections have been seen in meso-endemic and holo-endemic settings [35, 44, 45]. Similarly, based on *msp*-*1* genetic diversity marker, higher proportions of polyclonal infection have been seen in high endemicity settings, suggesting that malaria parasite polyclonality may be a useful proxy measure of level of endemicity [46]. Overall, genetic diversity was higher at the more malaria-endemic Ruhuha site than at Mubuga whilst 3D7 allelic families were more frequent than the FC27 families. At Ruhuha, 3D7 PCR products were 1.6-fold more than FC27 PCR products. Based on *msp*-*1*, similar observations of higher diversity at a holo-endemic site in Tanzania compared to hypo-endemic south-western Brazilian Amazon and meso-endemic southern Vietnam has been reported, with 3D7 reported as the most frequently circulating allele in this study [47].

The majority of *msp*-*2* FC27 alleles belonged to the 300–330-bp allele family while the most prevalent *msp*-*2* 3D7 allele belonged to the 200–300-bp allele family. Between the two sites, while the 300–330-bp allele was more frequent at Mubuga, the larger size (350–380, 400–450, 480–600) allelic families were more common at Ruhuha. In contrast to the FC27 gene, the 200–330-bp allele was the most frequent circulating allele at both Ruhuha and Mubuga. Of interest, findings from Mubuga of lower allelic diversity and lower frequency of circulating alleles point to a high likelihood of re-infection with the same allele. Differentiating between recrudescence and re-infection using *msp*-*2* in a low-endemic setting such as Mubuga may be limited by the *msp*-*2* low discriminatory power.

A number of factors, including an adequate sample size, use of validated genetic marker for diversity and allelic frequency, use of an automated gel reader to determine allelic family base pair sizes and a comparative analysis for the two study groups drawn from settings of different malaria transmission intensities, are major strengths of this study. However, there were some limitations. Firstly, there was a lack of earlier data on transmission intensity at either study sites to delineate local malaria endemicities. Secondly, being a cross-sectional survey design, study findings can only provide a baseline comparator for current diversity and disease clinical profiles but is unable to determine the value of diversity on other disease outcomes other than parasite density as well as time and impact of used intervention related effects. Thirdly, the study was done at two sites whilst in Rwanda, malaria risk is categorized into four malaria ecologic zones. Therefore, study findings may have limited generalizability, restricted to settings of comparable transmission and malaria control tool implementation levels. Fourthly, due to cost restrictions, we used a valid but lower discriminatory power assay (agarose gel electrophoresis) compared to other assays (e.g. capillary electrophoresis) and thus findings may be of a lower accuracy. Lastly, although *msp*-*2* is a validated molecular marker of diversity, use of one marker may miss variations at other polymorphic loci and underestimate the real magnitude of diversity.

## Conclusion

This study demonstrated a differential distribution in demographics, measured temperature, *P.falciparum* parasite density and genetic diversity as well as allelic distribution between individuals from two sites of variable malaria transmission intensities. HE and mean MOI were higher among isolates collected from the higher malaria Ruhuha site. Locally, characterizing malaria disease severity, based on clinical features and parasitaemia levels, across populations from settings of differing malaria transmission intensities is important in profiling malaria risk maps and in decision-making on which control tools may have optimal impact. The difference in diversity may have differential effects on multiple parameters including drug-resistant profiles, immunological responses to anti-malarial drug and effectiveness of vaccines tested in Rwanda in the future.
